# Effects of season and sex on the concentrations of fecal glucocorticoid metabolites in captive and free-ranging endangered mountain gazelles (*Gazella gazella*)

**DOI:** 10.3389/fvets.2024.1386009

**Published:** 2024-06-05

**Authors:** Mina Cansu Karaer, Tolga Kankılıç, Çağatay Tavşanoğlu, Marko Cotman, Nina Čebulj-Kadunc, Alenka Dovč, Tomaž Snoj

**Affiliations:** ^1^Food and Agriculture Vocational School, Çankırı Karatekin University, Çankırı, Türkiye; ^2^Institute of Science, Hacettepe University, Ankara, Türkiye; ^3^Division of Ecology, Department of Biology, Hacettepe University, Ankara, Türkiye; ^4^Department of Biology, Sabire Yazıcı Faculty of Science and Letters, Aksaray University, Aksaray, Türkiye; ^5^Institute of Preclinical Sciences, Veterinary Faculty, University of Ljubljana, Ljubljana, Slovenia; ^6^Clinic for Birds, Small Mammals and Reptiles, Veterinary Faculty, University of Ljubljana, Ljubljana, Slovenia

**Keywords:** mountain gazelle (*Gazella gazella*), fecal glucocorticoid metabolite concentrations, adrenocortical response, biological cycle, sex determination

## Abstract

**Introduction:**

The aim of our study was to measure fecal glucocorticoid metabolite (FGM) concentrations in captive and free-ranging male and female mountain gazelles (*Gazella gazella*) during their circannual cycle. In addition, FGM concentrations were used to track the intensity of the adrenocortical response in mountain gazelles during the same period.

**Methods:**

Fecal samples were collected from the ground in the Hatay Mountain Gazelle Wildlife Development Area in the Hatay Province of Türkiye (36°32’ N, 36°32′ E) in each season of the year (December, April, July, September). The sex of the animals was determined by detecting the *SRY* gene of the Y chromosome in DNA isolated from the fecal samples. FGM was extracted from dried fecal samples with methanol, and its concentration was measured using a previously partially validated ELISA.

**Results and discussion:**

The results indicate that season is the most important factor explaining the variability in FGM concentrations in mountain gazelles. In animals of both sexes, the highest concentrations of FGM were observed in September. The values were significantly higher in the captive population, perhaps due to unpredictable stress. In July, FGM concentrations were low in both populations. As a result of the overall analysis across seasons, the comparison of FGM concentrations between captive and free-ranging animals revealed higher concentrations in captive animals only in September but not in other seasons, although higher concentrations have been previously reported for several wild captive species. Due to predation risk, the presence of offspring can be considered a critical point in the biological cycle for the welfare of free-ranging mountain gazelles, as suggested by the higher FGM concentrations in the free-ranging population in July. The high number of visitors could be a challenge for mountain gazelles in captivity, as indicated by higher FGM concentrations during September. Sex had no effect on the FGM concentrations of either population.

## 1 Introduction

The mountain gazelle (*Gazella gazella*) inhabits areas in Israel and Palestine, the Golan Heights (1), the Jordanian side of the Jordan Valley (2) and the Hatay Province of Türkiye (3, 4). The population of mountain gazelles in Türkiye is isolated from other populations. The first data on population size in Türkiye reported approximately two hundred individuals in 2008, while the most recent inventory studies showed that there were 1,387 individuals in 2024 (data were estimated by the Hatay Branch of Nature Conservation National Parks). For the Israeli population of the species, approximately 5,000 individuals were counted in 2020 based on data from Israel’s Nature and Parks Authority (1). The species is classified as endangered by the International Union for Conservation of Nature and Natural Resources (5).

When a species is declared endangered, it calls upon human society, nature conservation policy, and the veterinary profession to ensure its survival in its natural habitat. Every individual holds significance in the conservation of endangered species, necessitating efforts to protect injured animals and motherless offspring while prioritizing their care, treatment, and conservation. The veterinary profession, with its focus on animal welfare, plays a fundamental role (6), utilizing assessments of HPA axis activity as a crucial indicator (7). Physiological data are essential for maintaining species viability and population health and for accurately assessing ecological outcomes. The incorporation of measures of adrenocortical activity, such as fecal glucocorticoid metabolite (FGM) concentrations, can aid in understanding animal behavior, disease susceptibility, and population performance, guiding effective conservation strategies globally (8).

The assessment of adrenocortical activity in mountain gazelles is important from both general and intraspecies-specific perspectives. In general, seasonal fluctuations in adrenocortical activity may indicate critical points for animal welfare when external (environmental) and internal (physiological) factors are considered together. At the intraspecific level, the comparison of the seasonal adrenocortical activity between the sexes provides information on sex-dependent differences in the adrenocortical response during the annual biological cycle, adding to the knowledge of mountain gazelle physiology. Intraspecies differences can also be detected between free-ranging and captive populations, as captivity has been recognized as a risk factor for distress in various species (9).

As the mountain gazelle population in Türkiye is isolated from larger populations in Israel (and Jordan), its survival is an important challenge for relevant local authorities and specialized experts involved in conservation policy, such as conservation and environment scientists, wildlife veterinarians, conservation educators and journalists. Therefore, knowledge of the animals’ habitat, potential risk factors, identification of critical points and assessment of animal welfare is important to ensure basic living conditions. In light of species conservation, identifying environmental stressors and limiting their harmful effects play important roles. Well-known examples of the stressors that affect species are macroenvironmental factors such as climate change (10), weather conditions, habitat fragmentation and loss due to traffic and agricultural activities (11–13), and pollution due to environmental pollutants (14), including sound (15) and light pollution (16). At the microenvironmental level, gazelle populations are faced with predation risk, species-specific infections, parasite infestations, and cohabitation and competition with domestic livestock. Despite some studies on genetics (4, 17), parasite infestation (18, 19), population status and distribution (20), and appropriate conservation planning (21), mountain gazelles are attractive and endangered mammal species and remain one of the least studied species in Türkiye. Therefore, studies addressing metabolic, endocrine and reproductive characteristics are essential to ensure the survival of this species in Türkiye. The adrenocortical response, as a basic adaptive mechanism, is not well known, so information in this area is needed to elucidate the critical points in the biological cycle of animals.

Our study thus aimed to understand the seasonal patterns in adrenocortical activity in mountain gazelles. Based on several publications showing seasonality of cortisol synthesis activity in different animal species (22–28), we assessed circannual fluctuations in HPA axis activity by measuring FGM concentrations in free-ranging and captive mountain gazelles of both sexes. The sampling schedule included a whole-year period that allowed the biological cycle of the species to be accounted for. Since captivity is a risk factor for animal distress (9), we also compared the adrenocortical response of captive and free-ranging mountain gazelles using FGM concentrations. In view of the abovementioned findings on adrenocortical activity, seasonal differences in FGM concentrations are expected. When comparing captive and free-ranging animals, a more intense adrenocortical response is expected in the captive population.

## 2 Materials and methods

### 2.1 Study species

The mountain gazelle, a member of the genus Gazella (Antilopini, Bovidae), has a lifespan of 8 to 12 years (29). Females can become pregnant at 18 months but under favorable conditions as early as 6 to 12 months (30). Males reach reproductive maturity at 3 years or as soon as 15 to 20 months with good nutrition. They typically give birth to one offspring per year after a 180-day gestation (31). The offspring, weighing approximately 2 kg, start on solid foods after 3 to 6 weeks and are weaned by 3 months (32). The mating season of the studied population of *G. gazella* is between December and January (Mina C. Karaer and Tolga Kankılıç, pers. obs.). During the mating season and early gestation, female groups tend to be larger, and female group home ranges correspond to the territory of adult, territorial males (33, 34). Although there is a dominant male in each mating group (35), other males attempt to mate with the females in the group. However, the dominant male usually seeks to prevent these matings. After the mating season, mating groups typically start to spread out in late January (Tolga Kankılıç, pers. obs.), with females remaining together (matrilineal groups with a mother and her last born daughters) and completing the pregnancy process (36). This social organization mirrors the structure observed in female *G. arabica* groups (36). The fawning season falls between May and June. Considering the mating season, June, July, and August are subsequent lactating periods, as lactation lasts for approximately 3 months. During this period, most females form matrilineal groups (36). Adult males are territorial and solitary throughout the year (35). However, nonterritorial males live in bachelor groups, comprising yearlings and adult males without a territory (34). After the lactating period, female fawns always remain in the matrilineal groups, whereas male fawns leave this group at approximately 6 months of age (30). We refer to this period beyond the reproductive cycle as the nonreproductive period. During this period, females and males have no attempt to mate; they are not organized into mating groups, and females are neither pregnant nor lactating.

### 2.2 Study area

The study was conducted in the Hatay Mountain Gazelle Wildlife Development Area, located in Hatay Province, Türkiye, close to the Syrian border (36°32’ N, 36°32′ E, 200–450 m.a.s.l.). The mountain gazelle habitat that was included in our study covered 13,228 hectares, including grassland vegetation with a few patches of shrublands, large expanses of croplands, and rocky hills around the Kırıkhan district of Hatay Province in Türkiye. The estimated population of mountain gazelles in this area was 1,331 individuals in 2023 (data from the Hatay Branch of the General Directorate of Nature Conservation and National Parks). This habitat is actively used by the species throughout the year, and it is the only habitat known for mountain gazelles in Türkiye (17). The protected area comprises a free-ranging population as well as a small captive population in the Hatay Mountain Gazelle Production Center. Most of the Hatay Mountain Gazelle Wildlife Development Area falls within a strictly controlled military zone. For this reason, entry to the free-ranging population area is prohibited for everyone except military personnel and farmers.

The free-ranging and captive populations are subjected to the same macroenvironmental conditions. Thus, the photoperiod and climatic conditions (Table 1) were the same for both populations (21). The vegetation structure is similar in both areas, i.e., in the free-ranging and captive populations. The dominant plant species in the study area included *Abutilon indicum, Sarcopoterium spinosum, Ziziphus jujuba, Zizyphus lotus, Plantago cretica, Asphodelus aestivus,* and *Arum dioscoridis* (21).

**Table 1:** Climatic conditions during the sampling periods in Hatay (the data are based on the Turkish State Meteorological Service data).

	Average temperature (°C)	Average humidity (%)	Total precipitation (mm)
December 2022	11.9	72.4	42.2
April 2023	18.5	54.3	21.6
July 2023	32.8	38.7	0.0
September 2023	28.7	50.1	0.6

However, at the microenvironmental level, some conditions differ among populations (Table 2).

**Table 2:** Human-associated activities and environmental conditions in captive and free-ranging populations of the mountain gazelle in Hatay province, Türkiye.

	Population	
Influence	Captive	Free-ranging
Anthropogenic		
Contact with humans	Constantly	Occasionally
Presence of visitors	Constantly (the number of visitors depends on the season)	Never
Direct contact with human	Constantly	Rarely
Hunting by humans	Never	Never
Concentrated feed	Constantly	Never
Use of parasiticides	Never	Never
Veterinary care	Irregularly	Never
Agricultural		
Access to the agricultural area	Never	Constantly
Domestic herbivore competitors	Never	Constantly
Environmental		
Hunting by predators	Rarely	Occasionally
Wild herbivore competitors	Never	Constantly
Roadkill	Never	Never
Access to water	Constantly	Depending on weather
Forage feed (pasture)	Depending on season	Depending on season

The free-ranging population thus feeds to a small extent on agricultural areas, especially during cultivation periods, as there are many agricultural areas and olive groves (21). The access to water for the free-ranging population is limited. There are only artifactitious ponds and water troughs in the field; however, during our fieldwork, we observed that water troughs and a small pond were dry for three seasons (April, July, and September 2023). Furthermore, the free-ranging population is being increasingly impacted by habitat loss due to agricultural activities.

Captive mountain gazelles are located at the Hatay Mountain Gazelle Production Center, which is located within a 12-ha fenced site within the research area. During sampling, there were 39 females and 11 males within a 12-ha region. The Hatay Mountain Gazelle Production Center was established to support the existing small population and to reintroduce mountain gazelles to suitable areas where they were previously distributed. However, this goal has not yet been achieved. The center also cares for injured animals and newborn gazelles abandoned by their mothers. Animals from the Center are not released back into the wild because they were habituated to humans, particularly the centers’ care staff. In this area, members of the captive population reproduce, and the offspring remain with their parents, continuing to inhabit the same area without separation. In rare but necessary circumstances (injuries, infections, etc.), veterinary care is provided. Other than these unique situations, there is no veterinary care. For captive individuals, animal care staff provide additional fodder. The captive population has feeding and watering troughs year-round. Due to the presence of fences, the area is partially closed to animal entry; however, animal care staff have observed red foxes occasionally breaching the fence. Additionally, hundreds of visitors visit this center every month.

### 2.3 Sampling

Our sampling schedule included all seasons that matched the biological cycle of *Gazella gazella.* Field sampling was performed in December 2022 (mating period), April 2023 (gestation period), July 2023 (time of birth and lactation period) and September 2023 (nonreproductive period). We collected 262 fecal samples in total. The number of collected samples in each season is shown in Table 3.

**Table 3:** Number of female and male samples included in the study during the sampling periods in the captive and free-ranging populations.

	Sampling period			
Population/sex	December	April	July	September
Females				
Free-ranging	21	20	28	29
Captive	2	6	13	14
**Total**	23	26	41	43
Males				
Free-ranging	12	19	26	31
Captive	7	14	12	8
**Total**	19	33	38	39

The numbers are based on the *SRY* gene detection results.

Before the fecal samples of mountain gazelles were collected from the ground, each individual was observed with binoculars from a minimum distance of 500 m for the free-ranging population and at a distance of 50 m for the captive population. When a group or individual defecated, the area was visually inspected (either by car or on foot), and fecal samples were collected as quickly as possible. Approximately 7 g of each fecal sample was collected from the ground and stored in polypropylene tubes, which were kept in a cooler (+4°C) during transportation to the laboratory.

### 2.4 Sex determination

The sex of the animals was determined by *SRY* gene detection in the DNA isolated from the fecal samples. Out of the 262 analyzed fecal samples, 133 were from females and 129 were from males (Table 3; Supplementary material). The results show the expected proportions and enable the formation of the animal groups studied.

For the preextraction procedure, all the samples were kept at −20°C. Before DNA extraction, each sample was weighed individually on a precision scale of 180–220 mg and transferred to 2 mL Eppendorf tubes with sample numbers written on them. Following preparation, the DNA extraction process was carried out, and genomic DNA was extracted from all 262 fecal samples. DNA was isolated using the QIAmp Fast DNA Stool Mini Kit (Qiagen, Hilden, Germany) according to the manufacturer’s instructions. After extraction, to detect sex, we used the *SRY* gene, which is the Y chromosomal gene that determines sex (37). PCR products were amplified in a 13.25 mL volume containing 11.25 mL of Platinum Supermix (Platinum PCR 2X MM; Invitrogen, Waltham, United States), 0.5 μL of each primer (diluted 10 pmol/μL) and 1 mL of template DNA. DNA was amplified via PCR using the following primers: SRY-BF (5′-GCA GCT GGG ATA TGA GTG GAA AAG-3′) and SRY-BR (3′-GCA GCT GGG ATA TGA GTG GAA AAG-5′). Primers flanking a target ruminant SRY gene were designed based on the NCBI GenBank reference assembly using Primer3 (38). PCR was performed using a SimpliAmp™ Thermal Cycler (Applied Biosystems, Waltham, United States) in a three-step process: first, denaturation for 10 min at 94°C, followed by 35 cycles of 30 s at 94°C, 30 s at 53°C, 1 min at 72°C, and finally, elongation for 10 min at 72°C. The PCR products were visualized by gel electrophoresis (2% agarose, TAE buffer). The expected amplicon size was 220 bp. We considered the presence of a band in a PCR result to be indicative of the sample belonging to a male; if no band was visible for the PCR product, we regarded this as indicating that the sample belonged to a female.

### 2.5 Measurement of the FGM concentrations in fecal samples

The extraction of FGM from fecal samples was performed according to previously described procedures (39–41) with slight modifications. The extraction was performed following this protocol. Approximately 2 g of each fecal sample was dried at 50°C for 24 h. A total of 0.5 g of the dry fecal sample was weighed and placed into a plastic tube. Five millilitres of absolute methanol was added, and the mixture was vortexed for 15 s and horizontally shaken at 300 rpm for 30 min. The extracts were centrifuged for 15 min at 4°C and 3,500 × g, after which two millilitres of the extract was added to a 4 mL tube. The extracts were dried under a stream of nitrogen at 40°C (nitrogen evaporator N-EVAP, Organomation Associates INC., Berlin, United States), and the dried extracts were dissolved in 1 mL of PBS. The dissolved samples were centrifuged for 15 min at 4°C and 3,500 × g. The supernatants were pipetted into 2 ml Eppendorf tubes and stored at −20°C until analysis.

The FGM concentrations in the fecal extracts were measured using a commercial ELISA kit (Demeditec, Kiel, Germany; ref. DES6611) following the manufacturer’s instructions. The absorbance was measured at 450 nm using a Multiskan FC microtiter plate reader (Thermo Fisher Scientific, Waltham, United States). The results obtained in ng FGM per mL extract were converted to ng FGM per g dry feces.

Prior to the measurement of the FGM concentrations in the fecal samples, partial analytical validation of the measurement procedure was performed. The validation included the calculation of intra- and interassay coefficients of variation (CVs) and the determination of recovery rates. Two samples of fecal extracts were run 16 times in one assay and repeated 4 times in the next assay. The recovery rate was determined by adding two known amounts of hydrocortisone (Sigma–Aldrich, St. Louis, United States) to two fecal samples before extraction. The FGM concentrations were also determined in these two fecal samples. The intra- and inter-CVs for the first sample were 8.72 and 13.95%, respectively, and those for the second sample were 7.62 and 12.20%, respectively. The recovery rates for the two samples were 88.6 and 85.2%, respectively. The data on the specificity of the method are given by the manufacturer of the ELISA kit. The cross-reactivity with other steroids was as follows (in %): testosterone <0.1, corticosterone 6.2, cortisone 0.8, 11-deoxycorticosterone 2.6, 11-deoxycortisol 50, dexamethasone <0.1, estriol <0.1, estrone 0.1, prednisolone 100, prednisone 0.9, progesterone <0.1, 17-hydroxyprogesterone 1.3, danazole <0.1, pregnenolone <0.1, estradiol <0.1, androstenedione <0.1. The abovementioned 11-deoxycortisol is one of the main FGMs (42). The range of the assay was between 1 and 300 ng/g.

In addition, biological evaluation of the FGM measurements was performed as demonstrated by Palme (7). The same fecal samples used for measurements of the FGM concentration in this study were also used to measure the fecal progesterone metabolite concentrations (Table 4). In April and July, we found high fecal progesterone metabolite concentrations (more than 4,000 ng/g) in some females. At this time, females are at the last phase of pregnancy, and high progesterone metabolite concentrations are most likely the result of pregnancy. Since females in the last stage of pregnancy are known to have elevated blood cortisol concentrations, we used 4 samples with high fecal progesterone metabolite concentrations for the biological evaluation of FGM measurements. In addition, we used four male samples collected in April with low fecal progesterone metabolite concentrations. Since April is not a mating season, the males remain in small groups or are solitary, which is why we expected lower FGM concentrations, as in pregnant females.

**Table 4:** Biological evaluation of the fecal glucocorticoid metabolites.

Animal sex	Fecal progesterone metabolites (ng/g)	Fecal glucocorticoid metabolites (ng/g)	Season of sampling / population
F	>4,000	94.7	July / free-ranging
F	>4,000	53.7	April / captive
F	>4,000	56.2	April / free-ranging
F	>4,000	75	April / free-ranging
M	410	7.4	April / free-ranging
M	641	10.5	April / free-ranging
M	330	6.5	April / free-ranging
M	337	13.8	April / free-ranging

### 2.6 Statistics

To analyze the effects of sex (male versus female), season (four biological periods), and population (free-ranging versus captive) on FGM concentrations in *G. gazella* individuals, we performed analysis of variance (ANOVA) considering these factors as fixed factors. In this analysis, we tested each factor and the interactions between factors against a null model to obtain F and *p* values. In the case of the season, a Tukey HSD *post hoc* test was also performed to assess significant differences between seasons. The FGM concentration data were log-transformed before the analyses to ensure normality and heteroscedasticity. Additional analyses were also performed to determine the separate effects of sex on FGM concentrations by performing two linear model analyses for each sex, in which season and population were included as fixed factors. All the statistical analyses were conducted in the R environment (43).

## 3 Results

**Figure 1:**
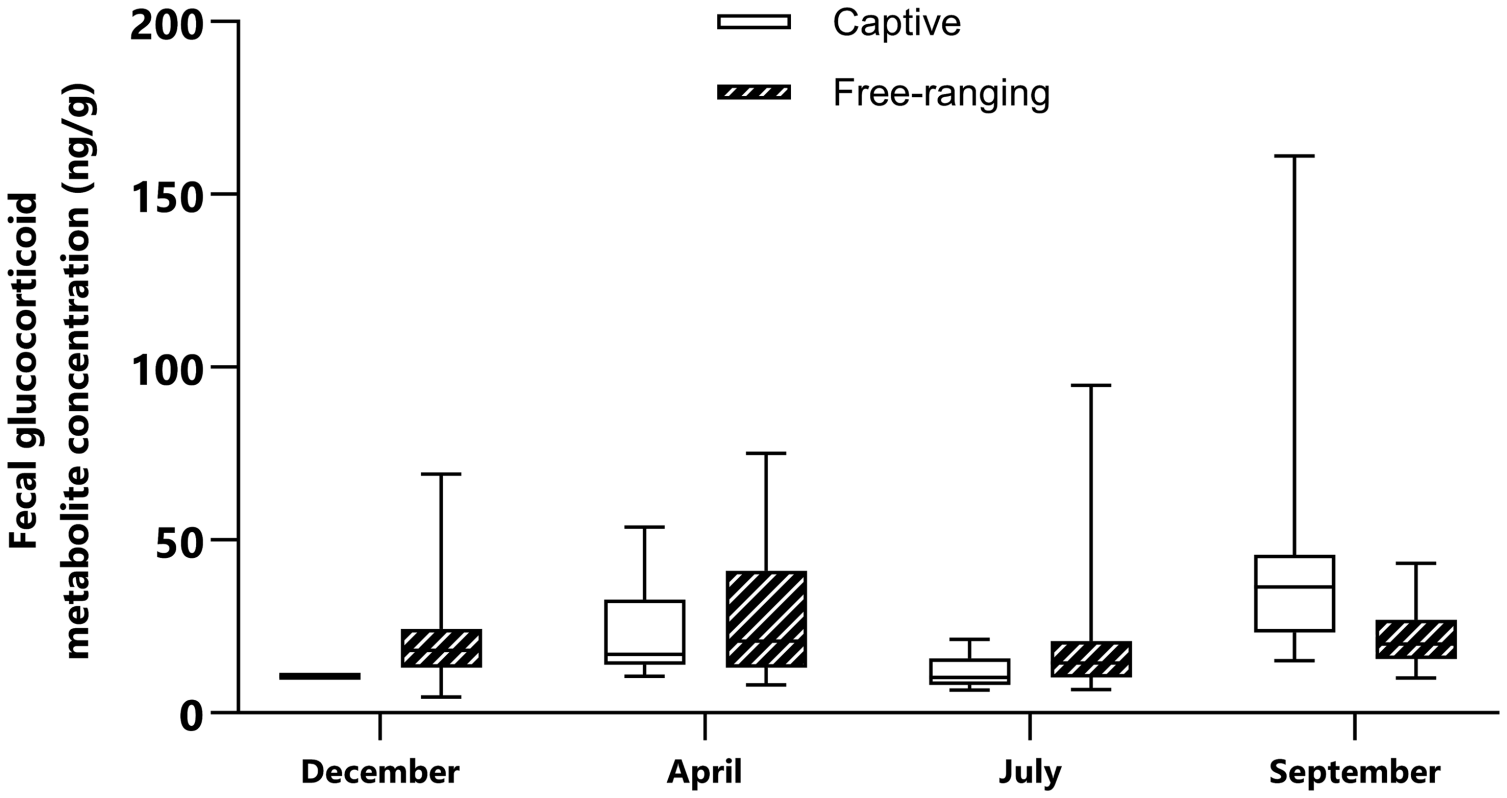
Median and interquartile range of the fecal glucocorticoid metabolite concentrations in captive and free-ranging female mountain gazelles (*Gazella gazella*) during different seasons.

**Figure 2:**
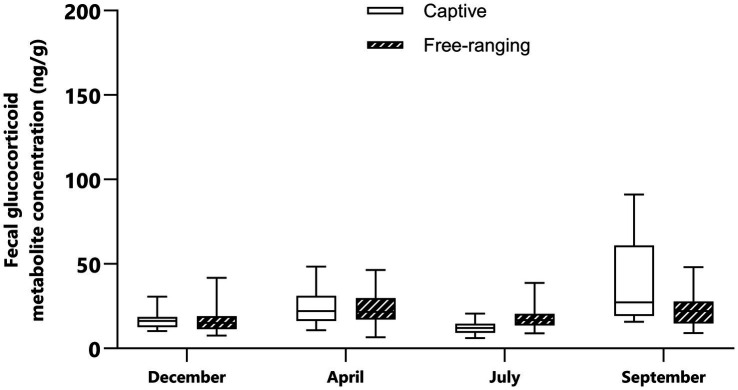
Median and interquartile range of the fecal glucocorticoid metabolite concentrations in captive and free-ranging male mountain gazelles (*Gazella gazella*) during different seasons.

There was a statistically significant difference in FGM concentrations among seasons (*p* < 0.0001); however, the FGM concentrations did not significantly differ between sexes or between captive and free-ranging populations (*p* > 0.05). When season was accounted for, however, the interactions between sex and population with season became statistically significant (*p* < 0.0001; Table 5). These results indicated that season is the most important factor explaining the variability in FGM concentrations in mountain gazelles. Additionally, we also found a significant difference in FGM concentrations between free-ranging and captive individuals in September (*p* = 0.0003) and a slight difference in FGM concentrations between these populations in July (*p* = 0.061). Notably, the difference in September was especially due to the increased FGM concentrations in females (*p* = 0.016) but not in males (*p* = 0.173; Figures 1, 2).

**Table 5:** Summary of the linear model analysis testing the effect of sex, season, and population on the fecal glucocorticoid metabolite concentrations in *Gazella gazella*.

Factor	d.f.	F	*p*
Sex	1	0.02	0.883
Season	3	15.58	<0.0001
Population	1	0.33	0.564
Sex*Season	7	6.73	<0.0001
Sex*Population	3	0.17	0.915
Season*Population	7	11.52	<0.0001
Sex*Season*Population	15	5.50	<0.0001

The results were obtained by comparing each factor or factor interaction against a null model.

There were significant differences in FGM concentrations among female gazelle seasons (Figure 1), specifically between September and July (*p* < 0.0001), between September and December (*p* = 0.032), and between April and July (*p* = 0.012). Among the captive female animals, the highest concentration was found in September, which was significantly higher than that in December (*p* = 0.048) and July (*p* < 0.0001). However, there was no significant difference in the FGM concentrations in free-ranging females among the seasons (*p* > 0.05).

There were significant differences (*p* < 0.05) in FGM concentrations between male gazelle and female gazelle in the same seasons. In captive male animals, the highest concentration was found in September, and this value was significantly higher than the values in December (*p* = 0.046) and July (*p* < 0.0001). In free-ranging males, however, there was no significant difference in the FGM concentrations between seasons (*p* > 0.05) (Table 6). The raw data on the FGM concentrations are shown in the Supplementary material.

**Table 6:** Summary of the linear model analysis testing the effect of season and population on the fecal glucocorticoid metabolite concentrations in *Gazella gazella* of both sexes.

Factor	d.f.	F	*p*
Females			
Season	3	8.10	<0.0001
Population	1	0.40	0.529
Season*Population	7	6.24	<0.0001
Males			
Season	3	7.54	0.0001
Population	1	0.02	0.892
Season*Population	7	5.42	<0.0001

## 4 Discussion

In this study, we aimed to gain insight into adrenocortical activity during the circannual cycle by measuring FGM concentrations in mountain gazelles. Two gazelle populations, free-ranging and captive, living under the same macroinveronmental conditions were investigated to determine how a population-specific microenvironment can influence their adrenocortical response. The assessment of FGM concentrations in mountain gazelles revealed seasonal fluctuations, as was also reported for other ruminants (24–27). Although several endogenous and exogenous factors influence adrenocortical response changes across seasons, it is difficult to identify the factors that are more important or less important for the adrenocortical response. We believe that the combination of all exogenous and endogenous factors that have a positive or negative effect on an individual can lead to adaptation processes that are controlled by the adrenocortical response. The well-being of an animal is ultimately a consequence of the sum of all negative and positive factors affecting it.

In the free-ranging population and captive population, lower FGM concentrations were detected in December than in April and September (Figures 1, 2). In December, vegetation provides sufficient high-quality forage feed for both populations. We also assumed that most females were at the beginning of their pregnancy at this time. Considering this, it seems that gazelles in December are not subjected to significant climatic and reproductive events, and their adaptation processes do not require a significant adrenocortical response. During this period, our study also revealed no significant differences in FGM concentrations between captive and free-ranging populations; thus, although there were some differences in the microenvironment (Table 2), these differences had no effect on the intensity of the adaptive processes assessed by FGM concentrations.

In April, the FGM concentrations were higher than those in December and July (Figures 1, 2). At this time, most of the females were in the 4th or 5th month of pregnancy. It is important to highlight that despite the lack of statistically significant differences in females between April and other studied seasons, many samples with higher FGM concentrations were identified during the analysis of April sample data. As supported by previous studies in wild ungulates, glucocorticoid concentrations increase in the late phase of pregnancy (44, 45). FGM concentrations are higher in the later phases of pregnancy because the production of glucocorticoids increases to meet the increased energy demand during intense fetal growth and because of intensive glucocorticoid synthesis by the fetus (46, 47). However, in some fecal samples, FGM concentrations were low, and these samples most likely represented early pregnant or nonpregnant females. When we compared the FGM concentrations of captive and free-ranging mountain gazelle in April, we did not observe a significant difference. Nonterritorial males from both populations form bachelor groups, while territorial males in each population live solitarily during this period. Since it has been reported that the adrenocortical response differs between territorial and nonterritorial males and that concentrations of fecal cortisol metabolites are higher in nonterritorial males (48), this could be the reason for the considerable variation in our data, the inconsistent results and the lack of significant differences.

In both populations, FGM concentrations were lowest in July compared to those in the other sampling periods. At this time of year, females and fawns stay in groups, while males remain solitary or in bachelor groups; however, some females are still pregnant. Although the ambient temperature was high in July, this apparently did not cause an intensive adrenocortical response. There are no published data on the thermoneutral zone of mountain gazelles, but it is likely that such environmental conditions are favorable for this species and that high temperatures do not cause heat stress. However, higher FGM concentrations were observed in the free-ranging population than in the captive population. The difference was very close to statistical significance (*p* = 0.061). The reason for this could be that weather conditions (Table 1) lead to limited food and water availability, which could be the reason for the slightly higher FGM concentrations in the free-ranging population. In contrast, the captive population has constant access to high-quality food and water, which may decrease the adrenocortical response of the animals. Furthermore, predators are more likely to exploit the presence of mothers with offspring, as they are less capable of detecting and avoiding predators, as has been reported for red deer *(Cervus elaphus)* (49), Przewalski’s gazelle *(Procapra przewalskii)* (50), Apennine chamois *(Rupicapra pyrenaica)* (51), fallow deer *Dama dama* (52) and roe deer *(Capreolus capreolus)* (53). In the captive population, fences provide protection and a safe place for mothers and fawns, which may also be reflected in lower FGM concentrations.

In September, FGM concentrations were found higher than those in the other seasons. In the captive population, FGM concentrations were high compared to those in other seasons (Figures 1, 2). According to research conducted on captive populations of *G. mohor*, *G. cuvieri* and *G. dorcas*, when *G. cuvieri* males were alone, higher glucocorticoid levels were detected, and they had lower glucocorticoid levels when they were with other males. *G. dorcas* and *G. mohor* males had higher glucocorticoid levels than females and lower values when they were alone (54). Therefore, it seems that the adrenocortical response depends on the species or even on the individual. Since we could not categorize and evaluate singleton individuals in the captive male populations, the reason for the high FGM concentrations measured in the captive populations in our study is not clear. In addition, the FGM concentrations in captive animals were significantly higher than those in free-ranging populations (*p* = 0.0003), since September is the month with the highest number of visitors. However, in the area of the captive population, the highest number of visitors was recorded in September. This could be a challenge and could be the reason for the high concentrations of FGM in the captive population.

**Figure 3:**
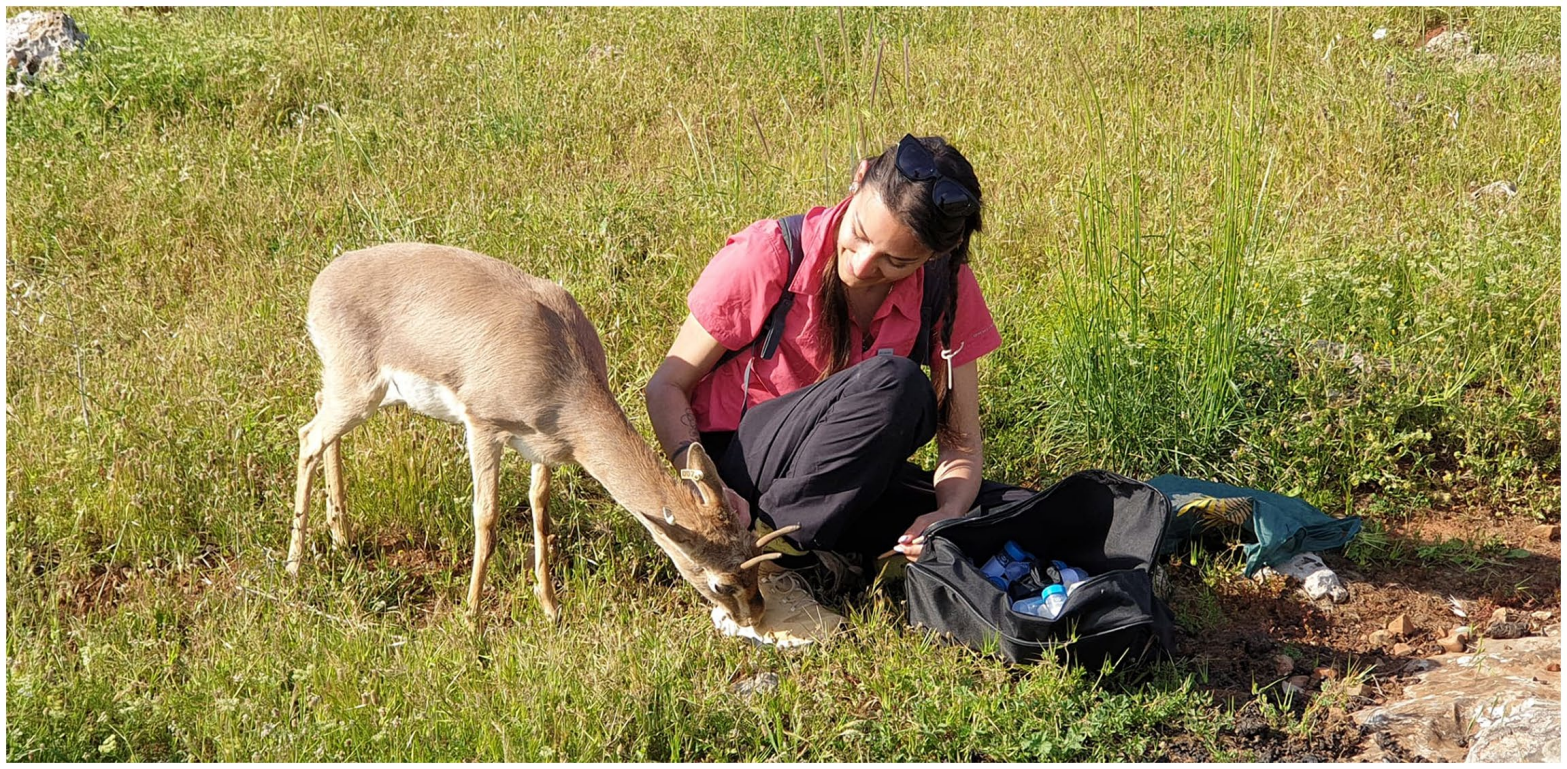
A female mountain gazelle, transferred to the Hatay Mountain Gazelle Production Center at an early age, was habituated to human presence.

When comparing captive and free-ranging populations, we generally expected higher FGM concentrations in captive animals, as several studies have shown similar results in some herbivores and carnivores (9), such as spotted hyenas (*Crocuta crocuta*) (55, 56), cheetah (*Acinonyx jubatus*) (57), fallow deer (*Dama dama*) (58, 59), and Canada lynx (*Lynx canadensis*) (60). The FGM concentration in captive animals could increase due to limited space, more socio-negative interactions, and the presence of humans. Our study revealed that the reactions of mountain gazelles are different. Compared to those in the free-ranging population, we found lower FGM concentrations in the captive population in December, April and July. We believe that for gazelle captivity offers several advantages, such as constant feed and water availability and protection from predators because of the protection provided by fences and animal care staff. As observed during fieldwork, some captive gazelles had no difficulties contacting humans, as some individuals were habituated and sought contact with humans (Figure 3), which has not been observed in free-ranging gazelles. It seems that contact with the care staff does not cause an intense adrenocortical response in captive mountain gazelles; on the other hand, groups of visitors may be the reason for this. Moreover, a constant supply of feed and water might have been particularly beneficial.

Since we were not able to track all the endogenous and exogenous factors that influence the response of the HPA axis, only internal factors, such as sex and the phase of the biological cycle, and external factors, such as food and water availability, intraspecific conflict, the presence of humans, human care and climate conditions, were considered and discussed. We are aware that the exact status of the animals examined is not known in detail. Although we have determined sex, several factors, such as age, health and reproductive status, as well as several external factors, remain unknown and have therefore not been accounted for when interpreting the results. We believe that these factors may also influence the activity of the HPA axis and could be a reason for the inconsistent data.

Although our study revealed seasonal variation in the adrenocortical response in mountain gazelles, there are some shortcomings that need to be addressed in future studies. For instance, our sampling schedule included only four samplings over 1 year. Consequently, the results do not provide insight into HPA axis activity and adrenocortical response between sampling periods. In addition, we did not identify the individual animals in our study, so age, health status, reproductive status, and social status were unknown. Future research should collect these data and take them into account when interpreting the results. Moreover, we conducted a partial analytical validation and biological evaluation of the FGM detection method for this study. However, for ethical and conservation reasons, pharmacological validation of the detection method was not performed, as it requires an ACTH challenge test and a dexamethasone suppression test (7), which cannot be performed on individuals of endangered species. However, a detailed analytical validation together with biological and pharmacological validation would increase the reliability of the detection method used.

In conclusion, FGM concentrations in mountain gazelles are subjected to seasonal fluctuations. The results suggest that the limited availability of food and water in summer can be regarded as a critical point for the free-ranging population. High visitor numbers can disturb the captive population. FGM concentrations do not differ between the sexes.

## Data availability statement

The original contributions presented in the study are included in the article/Supplementary material, further inquiries can be directed to the corresponding author.

## Ethics statement

Ethical approval was not required for the study involving animals in accordance with the local legislation and institutional requirements because the fecal samples were collected from the ground, there was no direct contact with the animals. The work was approved by the General Directorate of Nature Conservation and National Parks of Türkiye with a document no: 8037146, dated 08.12.2022. Written informed consent was obtained from the individual(s) for the publication of any potentially identifiable images or data included in this article.

## Author contributions

MCK: Conceptualization, Formal analysis, Investigation, Writing – original draft, Writing – review & editing. TK: Investigation, Supervision, Writing – review & editing. ÇT: Conceptualization, Formal analysis, Investigation, Writing – original draft, Writing – review & editing. MC: Investigation, Writing – review & editing, Methodology. NČK: Investigation, Writing – review & editing. AD: Formal analysis, Writing – review & editing, Data curation. TS: Formal analysis, Writing – review & editing, Conceptualization, Investigation, Writing – original draft.
